# Identification of a pro-protein synthesis osteosarcoma subtype for predicting prognosis and treatment

**DOI:** 10.1038/s41598-024-67547-z

**Published:** 2024-07-16

**Authors:** Chengfeng Yi, Jun Liu, Shibing Zhao, Deliang Gong, Bohan Xu, Ao Li, Erbao Bian, Dasheng Tian

**Affiliations:** 1 Department of Orthopaedics, The Second Affiliated Hospital of Anhui Medical University, Hefei, 230601 China; 2 Institute of Orthopaedics, Research Center for Translational Medicine, The Second Affiliated Hospital of Anhui Medical University, Hefei, 230601 China

**Keywords:** Osteosarcoma, scRNA-seq, Intra-tumor heterogeneity, Therapeutic target, Cancer, Computational biology and bioinformatics, Genetics, Oncology

## Abstract

Osteosarcoma (OS) is a heterogeneous malignant spindle cell tumor that is aggressive and has a poor prognosis. Although combining surgery and chemotherapy has significantly improved patient outcomes, the prognosis for OS patients with metastatic or recurrent OS has remained unsatisfactory. Therefore, it is imperative to gain a fresh perspective on OS development mechanisms and treatment strategies. After studying single-cell RNA sequencing (scRNA-seq) data in public databases, we identified seven OS subclonal types based on intra-tumor heterogeneity. Subsequently, we constructed a prognostic model based on pro-protein synthesis osteosarcoma (PPS-OS)-associated genes. Correlation analysis showed that the prognostic model performs extremely well in predicting OS patient prognosis. We also demonstrated that the independent risk factors for the prognosis of OS patients were tumor primary site, metastatic status, and risk score. Based on these factors, nomograms were constructed for predicting the 3- and 5-year survival rates. Afterward, the investigation of the tumor immune microenvironment (TIME) revealed the vital roles of γδ T-cell and B-cell activation. Drug sensitivity analysis and immune checkpoint analysis identified drugs that have potential application value in OS. Finally, the jumping translocation breakpoint (JTB) gene was selected for experimental validation. JTB silencing suppressed the proliferation, migration, and invasion of OS cells. Therefore, our research suggests that PPS-OS-related genes facilitate the malignant progression of OS and may be employed as prognostic indicators and therapeutic targets in OS.

## Introduction

Osteosarcoma (OS) is a heterogeneous malignant spindle cell tumor that most commonly occurs in children and adolescents^1,2^. It can arise in any bone but usually occurs in the metaphysis of long bones^3^. Surgery and chemotherapy, the standard treatment for OS established in the 1980s, has enabled long-term survival in 60% of patients with nonmetastatic tumors^4^. However, there are disadvantages to both chemotherapy and surgery. Excision by surgery frequently has a negative impact on athletic ability. Current chemotherapeutic drugs have low specificity, have serious adverse reactions, and are prone to drug resistance^5^. Tumors are composed of subpopulations (subclones) of cells of different phenotype due to the presence of ITH, which is also a powerful aid to cancer progression and therapeutic failure^6^. Over the past decade, the establishment of well-annotated tissue banks and the development of comprehensive molecular analysis techniques and preclinical models have enhance our understanding of the molecular mechanisms and biological heterogeneity of OS at the pathophysiological level^7–9^. However, progress in identifying new therapies has been slow, and treatment options are especially limited for patients with advanced or metastatic disease^10^. Thus, it is extremely valuable to study the molecular mechanisms and intra-tumor heterogeneity (ITH) related to the genesis and progression of OS, which may help us to filter out essential molecules or biomarkers for early diagnosis and targeted treatment.

Following an in-depth review of the scRNA-seq data, the current study identified seven unique subsets of OS cells based on ITH, each of which was annotated subjectively to facilitate further investigation. Next, the differentially expressed genes (DEGs) of each subtype were obtained, and a prognostic model was established based on the cell subtype with the worst survival indications. This cell subtype was annotated as pro-protein synthesis osteosarcoma (PPS-OS). Moreover, a nomogram was established by merging DEGs with clinical factors. Furthermore, the correlations of the model score with drug sensitivity and the TIME were assessed, thereby expanding the prognostic value of the gene signature for OS patients. In the final step, we conducted experiments in vitro to verify the effect of silencing the JTB gene. Our research suggests that PPS-OS-related genes play a vital role in the development and progression of OS and that JTB may be a novel target in the treatment of OS.

## Methods

### Data collection

The scRNA-seq data were obtained from the GSE152048 dataset in the Gene Expression Omnibus (GEO) database (https://www.ncbi.nlm.nih.gov/geo/, accessed on 15 January 2023). Eighty-five clinical case samples, including the survival information of OS patients, were obtained from the Therapeutically Applicable Research to Generate Effective Treatment (TARGET) database (https://ocg.cancer.gov/programs/target, accessed on 16 January 2023) and serve as the training cohort. Ninety clinical case samples from the GEO database were also gathered and served as the testing cohort. The samples (n = 396) for normal tissue were downloaded from the GTEx (https://gtexportal.org, accessed on 16 January 2023).

### Processing of the scRNA-seq data and annotation of cell clusters

The data were processed statistically by the Seurat package in R 3.6.3^11^. Firstly, the quality control of OS scRNA-seq data obtained from the database was performed, including correcting batch differences by using the Seurat3 software package^12^. The “LogNormalize” algorithm was employed to normalize the data before unsupervised clustering of cells, and dimensionality reduction and t-SNE were employed to visualize the data^13^. The SingleR package was used to annotate every cluster’s cell type^14^. The “FindAllMarkers” function in Seurat was employed to screen out differentially expressed genes (DEGs)^15^, and the criteria were set as follows: absolute log2-fold change (FC) ≥ 1, false discovery rate (FDR) < 0.05, adjusted P value < 0.05 (derived by Bonferroni’s multiple test correction). The cell clusters were annotated after subjective interpretation of marker genes and the outcomes of GO functional enrichment analysis.

### GO and KEGG analysis

The clusterProfiler R package was employed to implement GO^16^ functional enrichment analysis and KEGG^17^ pathway enrichment analysis. The GO results include multiple biological processes (BPs), cellular components (CCs), and molecular functions (MFs). The threshold for significant enrichment was set as P < 0.05.

### Development of a prognostic model based on PPS-OS-related genes

Analysis of gene differential expression and univariate cox regression analysis were implemented to filter genes linked to prognosis from the PPS-OS gene set using a p < 0.05 threshold. We used the LASSO regression algorithm in the “glmnet” R package to select the best genes and prevent overfitting. Simultaneously, the risk score for each OS patient was calculated as follows^18^:

$$Riskscore = \sum\limits_{x = 1}^{n} {coef(x) \times \exp (x).}$$

The symbols exp (x) and coef (x) represent the expression level and gene coefficient of gene X, respectively. The median risk score served as the division criterion, and samples of patients were categorized into low- and high-risk subgroups. The overall survival difference between the two risk groups was assessed by Kaplan–Meier (K‒M) survival analysis with the log-rank test^19^. A receiver operating characteristic (ROC) curve was employed to evaluate the sensitivity and specificity of the PPS-OS signature^20^.

### Nomogram construction and validation

Univariate and multivariate Cox regression analyses were performed to determine clinical parameters associated with prognosis and to derive their hazard ratio (HR). These parameters included gender, age, tumor site, metastatic status, and risk scores. Based on these clinical parameters, “rms” R software was used to plot the clinical nomogram^21^. In addition, calibration curves were used to assess how well the predicted and actual survival rates agreed with one another.

### Evaluation of the TIME

The stromal score, ESTIMATE score, immune score and tumor purity level of the entire sample were calculated through the “ESTIMATE” algorithm^22^. The degrees of infiltration of different immune cells in the two risk subgroups were obtained using the ssGSEA algorithm. The immunophenoscore (IPS) of OS patients derived from The Cancer-Immune Group Atlas (TCIA) (https://tcia.at/home) was evaluated using ggpubr R software. Effector cell (EC) score, immunosuppressive cell (SC) score, MHC molecule (MHC) score, and immune checkpoint (CP) score are the four categories that make up the IPS^23^. The IPS (range 0–10) was calculated using the gene expression in the corresponding cell type, and the score was proportional to the immunogenicity.

### Prediction of immunotherapy and chemotherapy

The tumor immune dysfunction and exclusion (TIDE) score and subclass mapping were used to estimate the clinical immune checkpoint effect on CTLA4 and PD-1 to reflect the tumor response to immune checkpoint blockade (ICB). The limma, ggpubr, and pRRophetic packages of R were used to screen potential chemotherapy drugs for OS.

### Cell culture and transfection

The human OS cell lines HOS, MG-63, 143B, R-1059D, U-2OS, and SAOS-2 were obtained from the American Type Culture Collection (US). MEM or DMEM containing FBS (10%, Gibco, USA) and penicillin‒streptomycin solution (100 µg/ml, Beijing Solarbio Science & Technology Co., Ltd.) was used as the culture medium. We discovered that research on JTB (jumping translocation breakpoint) in OS is scarce and has not been experimentally validated. Therefore, to target and knockdown JTB, we employed siRNA synthesized by General Biol (Anhui, China). When the cells were cultured to an optimal density in a 6-well plate, the culture medium was replaced, and siRNA was transfected using jetPrime reagent (Poly plus-transfection®). The sequences for the two JTB siRNAs were as follows: JTB-1-forward GGAAGAGUUUGUGGUAGCATT, JTB-1-forward UGCUACCACAAACUCUUCCTT, JTB-2-forward CAGCGACAAUUGGACAGAATT, JTB-2-forward UUCUGUCCAAUUGUCGCUGTT.

### RNA extraction and RT-qPCR

Total cellular RNA was isolated using TRIzol reagent (Invitrogen, Thermo Fisher Scientific), followed by detection of the quality and concentration of RNA with a Nanodrop spectrophotometer (IMPLEN GmbH). cDNAs were obtained by reverse transcription. Subsequently, RT-qPCR experiments were carried out with SYBR Green mix (TaKaRa Biotechnology, China). Expression levels of target genes were reported by the 2-ddCt method. The primer sequences were as follows: GAPDH forward: 5ʹ-CGCTCTCTGCTCCTCCTGT-3ʹ; reverse: 5ʹ-ATCCGTTGACTCCGACCTA-3ʹ. JTB forward: 5ʹ-AATAGGCAACTCCGGCCTTC-3ʹ; reverse: 5ʹ-AGAGGGACCTACTCCACAGG-3ʹ.

### Cell viability assay

The transfected cells (3 × 10^3^) were seeded in 96-well plates and incubated at 37 °C. After incubation for 24, 48, and 72 h, CCK-8 reagent (Kumamoto, Japan) was injected and incubated again for 4 h at 37 °C. The absorbance at 450 nm was detected by a microplate reader (Thermo Fisher Scientific).

### Migration and invasion assay

The assays were conducted in a 24-well plate utilizing a chamber insert with a pore size of 8 μm (3422, Corning, USA). For the migration assays, 2 × 10^4^ cells in serum-free medium were transferred to the upper chamber. The lower chamber was filled with culture media (600 μl) containing 30% FBS.

The invasion assay was performed the same as the migration assay, with a few exceptions: 1 × 10^5^ cells were seeded into the upper chamber, which had been precoated with Matrigel (356234; BD Biocoat). Later, they were all incubated for 24 h (migration assay) or 48 h (invasion assay) at 37 °C and 5% CO_2_. After incubation, cells were fixed and stained with 4% paraformaldehyde and 0.5% crystal violet. After the cells on the upper surface of the chamber were wiped away, the cells were photographed and counted under an inverted microscope.

### Western blotting

RIPA lysis buffer was used to extract total protein from 143B and HOS cells. The BCA Protein Assay Kit and SDS-PAGE were employed for protein content detection and protein separation. The proteins were transferred onto a PVDF membrane (Millipore Corp, USA), and the blots were blocked with TBST with 5% skim milk for 3 h and then incubated with the primary antibody overnight at 4 °C. Afterward, membranes were washed with TBST once per minute for a total of 3 times, and TBST was further diluted to a ratio of 1:10,000, followed by the addition of a second antibody and shaking for 1 h at room temperature. Afterward, immune complexes were detected using ECL reagent. The antibodies used in this experiment included anti-β-actin (Abcam, ab8226), anti-ZEB1 and anti-PCNA (Proteintech, China).

### Analysis of data

For all statistical testing and analysis during this investigation, GraphPad Prism 8 and R software were used. Continuous variables were compared using the Wilcoxon test. For correlation analysis, the Spearman correlation test was employed. The findings are presented in terms of the mean ± standard deviation (SD). The t test was employed to analyze intergroup differences. “NS” indicates P > 0.05, “*” indicates P < 0.05, “**” indicates P < 0.01, and “***” indicates P < 0.001. All experiments were repeated three times.

## Results

### Identification of 7 cell clusters

**Figure 1:**
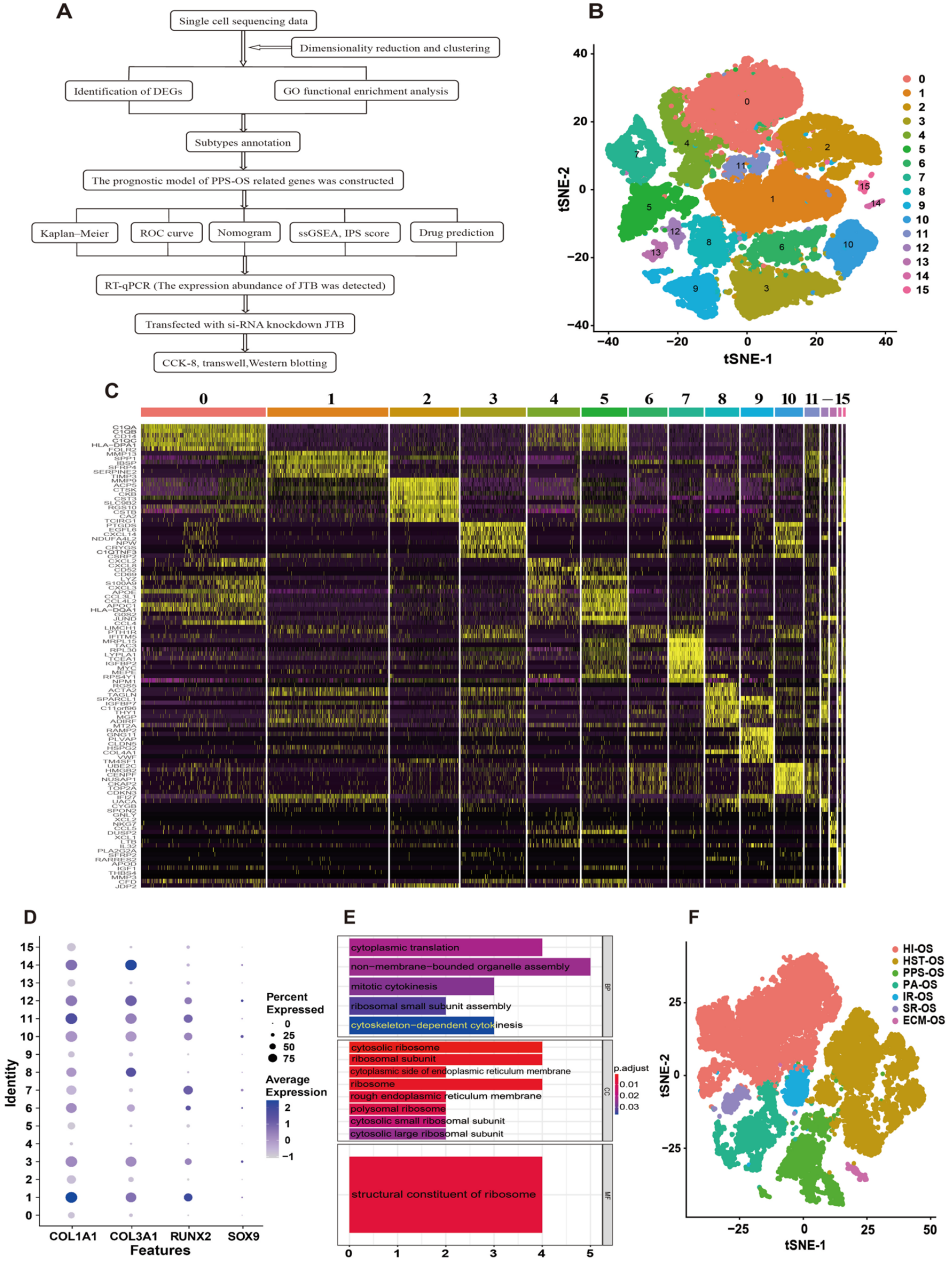
Flowchart and the single-cell transcriptomic analysis of OS tissues. (**A**) Flowchart of this study. (**B**) Unbiased clustering of the cells identified 16 main clusters in parallel according to their gene profiles and canonical markers. (**C**) Heatmap showing the DEGs of 16 cell subtypes. (**D**) The proportion of specific markers in 16 clusters and their scaled relative expression levels. (**E**) GO analysis of one cluster of PPS-OS. (**F**) The annotation of seven distinct cell subtypes of OS.

A schematic diagram for the research is displayed in Fig. 1A. After removing batch effects and performing an initial quality control assessment, the scRNA-seq data from public databases were used for further analysis. Unbiased clustering of the cells identified 16 main clusters based on t-SNE analyses (Fig. 1B, Supplementary Table 1). Figure 1C displays the average expression of DEGs of 16 clusters. The bubble plots compare the proportions and relative expression levels of specific markers in 16 clusters (Fig. 1D). The OS cells were extracted according to the marker genes (COL1A1, COL3A1, RUNX2, etc.) of malignant osteoblastic cells^24,25^ and divided into seven subtypes (Supplementary Table 2). Cluster 0 contained 412 genes; Cluster 1 contained 594 genes; Cluster 2 contained 370 genes; Cluster 3 contained 351 genes; Cluster 4 contained 259 genes; Cluster 5 contained 602 genes; and Cluster 6 contained 381 genes.

### Functional enrichment analysis and cell subtype annotation

The GO functional enrichment analysis revealed that cluster 2-correlated genes were mainly enriched in crucial factors or steps in protein synthesis. Figure 1E shows the biological processes (BPs), including ‘cytoplasmic translation’ (GO:0002181) and ‘ribosomal small subunit assembly’ (GO:0000028). The significantly enriched cellular components (CCs) included ‘cytosolic ribosome’ (GO:0022626), ‘ribosomal subunit’ (GO:0044391), and ‘ribosome’ (GO:0005840). Among the molecular function (MF) terms, the genes were enriched for ‘structural constituent of ribosome’ (GO:0003735) and ‘protein self-association’ (GO:0043621). The results of GO analysis of the remaining subtypes were detailed in the (Supplementary Fig. 1A–F). To facilitate valuable researches on the biological significance of each cell subtype, the biological functions and interactions of GO terms were analyzed, and annotated the subtype function as highly invasive osteosarcoma (HI-OS), homeostatic type osteosarcoma (HST-OS), pro-protein synthesis osteosarcoma (PPS-OS), pro-angiogenic osteosarcoma (PA-OS), immunoreactive osteosarcoma (IR-OS), stress-related osteosarcoma (SR-OS), and extracellular matrix-enriched osteosarcoma (ECM-OS) (Fig. 1F).

### Construction of a prognostic model containing six PPS-OS marker genes

**Figure 2:**
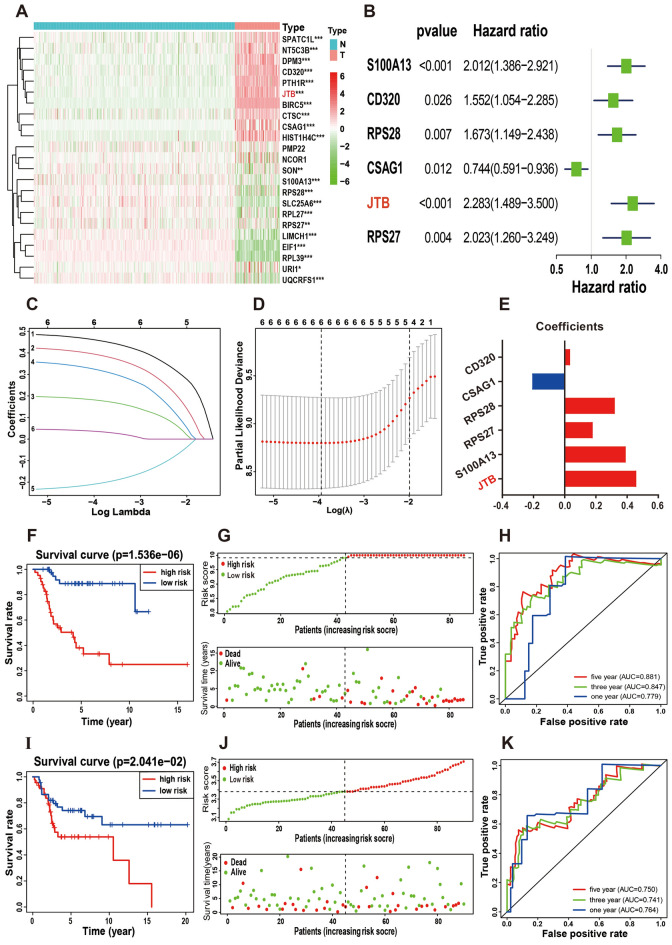
Establishment and validation of the risk prognostic mode. (**A**) The heatmap shows expression levels of 23 PPS-OS related genes in normal and OS tissues. (**B**) Univariate Cox regression analysis presents the hazard ratios and P-value of 6 PPS-OS related genes. (**C**) Obtainment of the optimal λ value. (**D**) The LASSO Cox analysis identified 6 genes apparently affecting the model. (**E**) The 6 risk signature genes and the corresponding coefficients. (**F,I**) The survival analysis in the training (**F**) and testing set (**I**). (**G,J**) The distribution of risk score as well as survival time for the two risk subgroups in the training (**G**) and testing set (**J**). (**H,K**) The predictive effificiency of the risk score is presented by the ROC curves in both training (**H**) and testing set (**K**).

The overall survival rates of different risk groups in every cell subtype were shown by K‒M curves (Fig. 2F, Supplementary Fig. 2A–F). The P values representing the significance of the survival differences are 0.0000015 for PPS-OS, 0.000097 for ECM-OS, 0.00014 for SR-OS, 0.00028 for HST-OS, 0.0035 for PA-OS, 0.0057 for HI-OS, 0.067 for IR-OS, respectively. The PPS-OS with the most significant survival differences (minimum P value) between the two risk groups was finally identified as the focus of this study.

Subsequently, 23 PPS-OS-related genes that were significantly associated with prognosis were extracted from 370 genes. In the TARGET and GTEx merged cohort, differential expression analysis was applied to compare the DEGs between the tumor and normal sets (Fig. 2A). Afterward, six genes for modeling were identified by univariate cox regression analysis, and the HR and P values of each gene were also calculated (Fig. 2B). Figure 2C,D shows that with the decline of log λ, the corresponding coefficient of the genes likewise diminished to 0, and finally, 6 genes in cross-validation were within the partial likelihood estimation bias minimum value. These genes (CSAG1, RPS27, RPS28, CD320, JTB, and S100A13) are significantly associated with overall survival and are potential prognostic genes. The risk coefficients for these six genes are shown in Fig. 2E.

**Figure 3:**
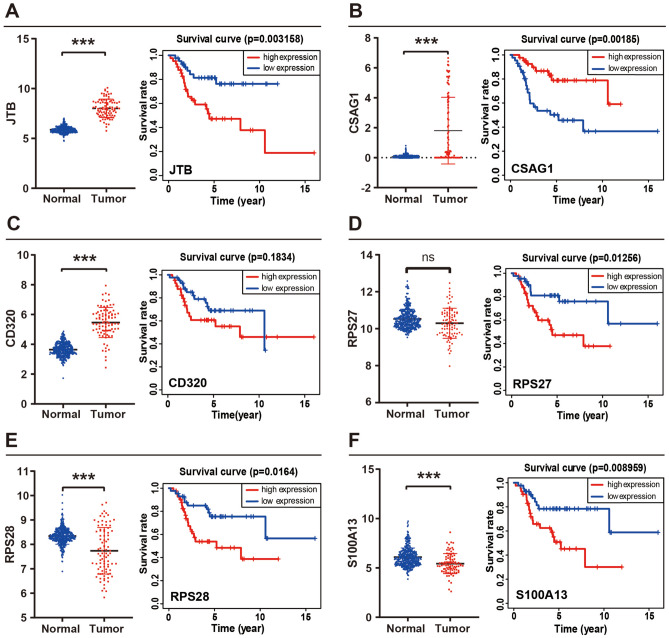
Expression levels and survival analysis of individual genes. (**A–F**) The expression level of individual gene between the normal and tumor groups, as well as the results of survival analysis (red represented high expression and blue represented low expression).

The median risk value (9.92) was used as the dividing point of the calculated risk score, and 85 patients in TARGET were categorized into low- and high-risk groups. Figure 2F shows a significant difference (P value = 0.0000015) between the two risk groups and a negative correlation between risk scores and patient survival time. The two-dimensional distribution of survival status as well as risk scores for the two risk groups are displayed in Fig. 2G and reflect the poorer survival rate in the high-risk group. Moreover, the areas under the curves (AUCs) for 1, 3, and 5 years were 0.779, 0.847, and 0.881, respectively (Fig. 2H), reflecting that the model has accurate and meaningful predictive capabilities. In the testing set, the survival curve (Fig. 2I), risk score and distribution of patient survival status (Fig. 2J), and AUC values (Fig. 2K) were similar to those in the training set. These results indicated that the prognosis model containing six PPS-OS marker genes can predict disease outcomes in OS patients. Figure 3A–F shows the expression of these six genes in the tumor and normal groups as well as the overall survival differences between the high- and low-risk groups.

### Assessment of the independent prognostic value of clinical factors

**Figure 4:**
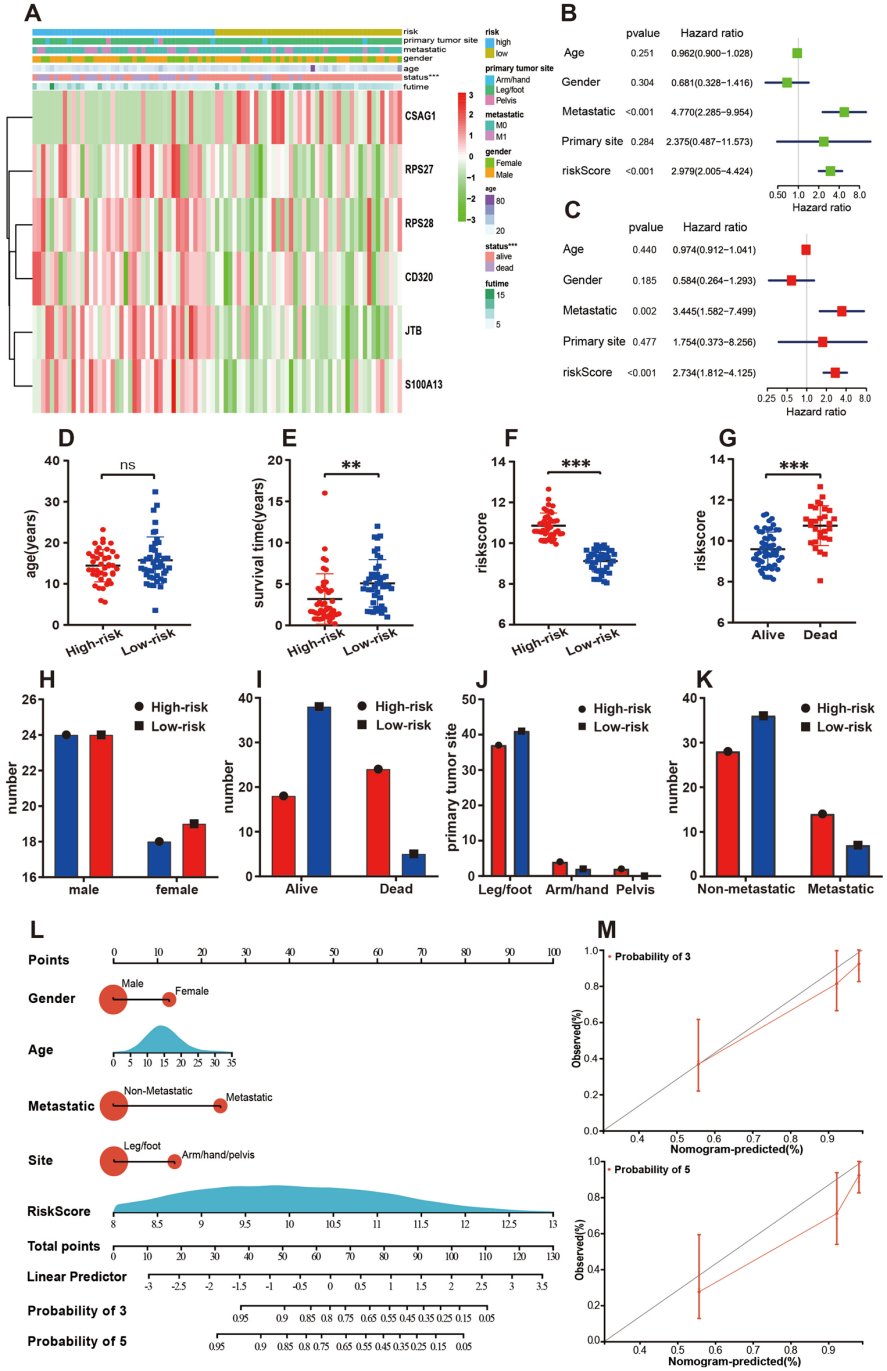
Relationship between the clinical variables in the TARGET dataset. (**A**) The heatmap displays the differential distribution of clinical variables and 6 PPS-OS related genes expression levels in low- and high-risk. (**B,C**) Univariate (**B**) and multivariate (**C**) cox regression analyses including age, gender, metastatic, primary tunmor site and risk score. (**D–K**) The various clinical variables were precisely compared one by one between the high- and low-risk groups. (**L**) A prognostic nomogram for predicting the overall survival of OS patients for the 3 and 5 years. (**M**) The calibration curves of the nomogram for the 3 and 5 years.

In analyzing the relationship between clinical variable information and prognostic value, we discovered significant differences in survival status, primary tumor site, tumor metastasis status and risk score between risk groups. The high-risk group had a shorter survival time, more diverse primary tumor sites (including the limbs and pelvis), and a higher rate of tumor metastasis (Fig. 4A). Figure 4B shows that the risk score (P < 0.001) and metastasis (P < 0.001) were independent factors affecting the prognosis of patients with OS, and multivariate Cox regression analysis generated comparable results (Fig. 4C). Figure 4D–K shows the relationship between various clinical factors in a more intuitive and accurate manner.

The nomogram we built is a highly reliable mathematical model. As shown in the nomogram, the most common age for OS patients was approximately 15 years old. Another concern was the tumor metastasis status. Distant metastatic tumors were clearly much more likely to cause death than localized tumors. The nomogram also predicted the survival rate of OS patients at 3 and 5 years in a systematic manner (Fig. 4L). Figure 4M shows that the calibration curve is relatively close to the 45° reference line, suggesting that the nomogram-predicted overall survival was consistent with actual overall survival.

### Comprehensive analysis of the TIME

**Figure 5:**
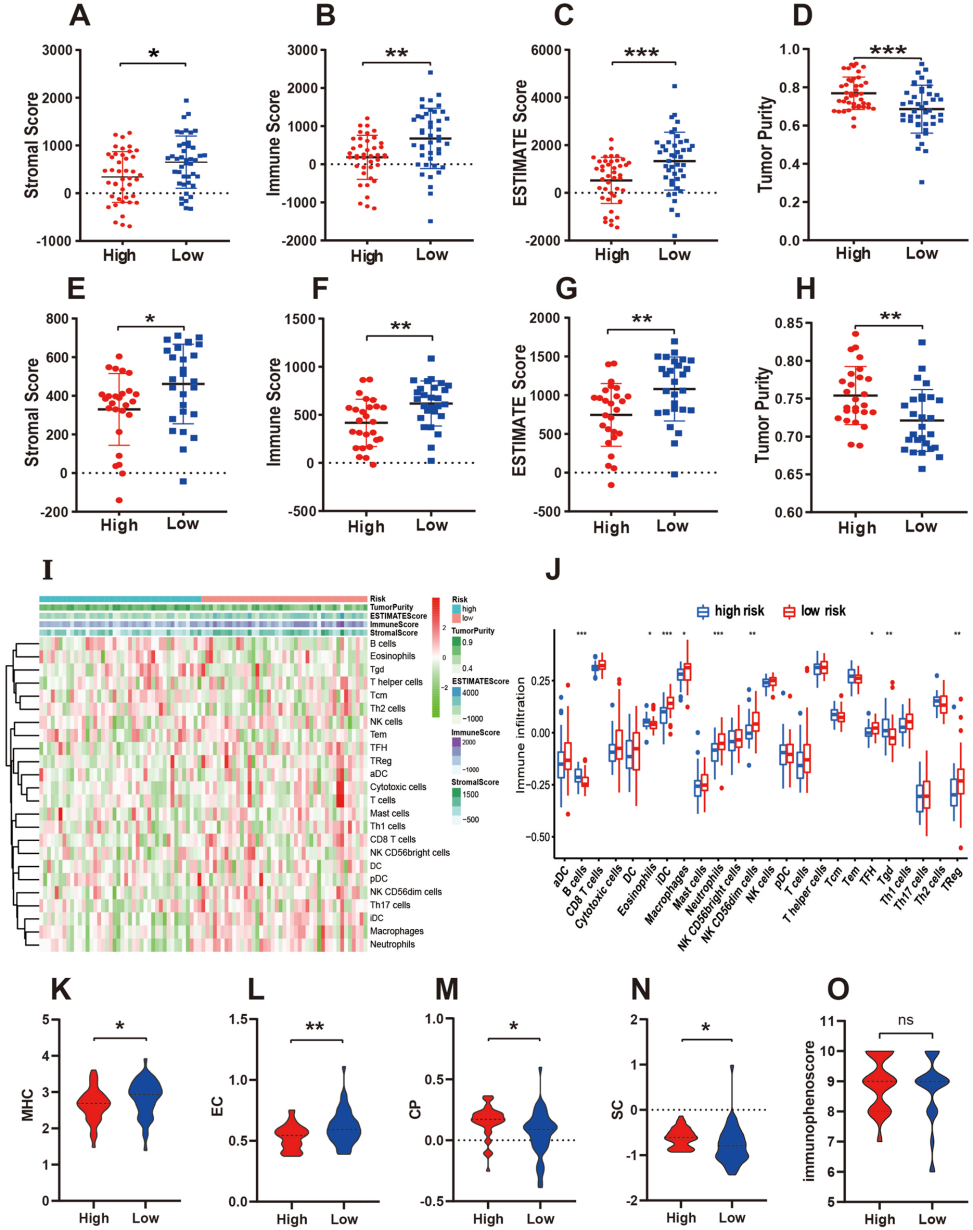
Analysis of tumor immune microenvironment. (**A–H**) The score of Stromal, Immune, ESTIMATE, and Tumor Purity between the two risk subgroups in the training (**A–D**) and testing set (**E–H**). (**I**) Heatmap showing the infiltration of 24 immune cells in the high- and low-risk groups. (**J**) Violin plots showing the difference in immune cell infiltration between the high- and low-risk groups. (**K–O**) The immunophenoscore analysis of two risk groups.

Analysis of the training (Fig. 5A–D) and validation sets (Fig. 5E–H) revealed that the high-risk subgroup had a lower immune score, stromal score, and ESTIMATE score and higher tumor purity. Figure 5I–J indicates that the infiltration of B cells and gamma delta T cells (Tgd, γδ T cells) was higher in the high-risk group. Figure 5K–N shows the differences between the four components of the IPS. We found that IPS was higher in both risk groups, and it represents OS as a more immunogenic tumor (Fig. 5O). Together, these results indicated that PPS-OS-related genes have a substantial link with immune infiltration, regulate multiple aspects of tumor immunity, and may play an essential role in the progression of OS.

### Prediction for immunotherapy and drug sensitivity analysis

**Figure 6:**
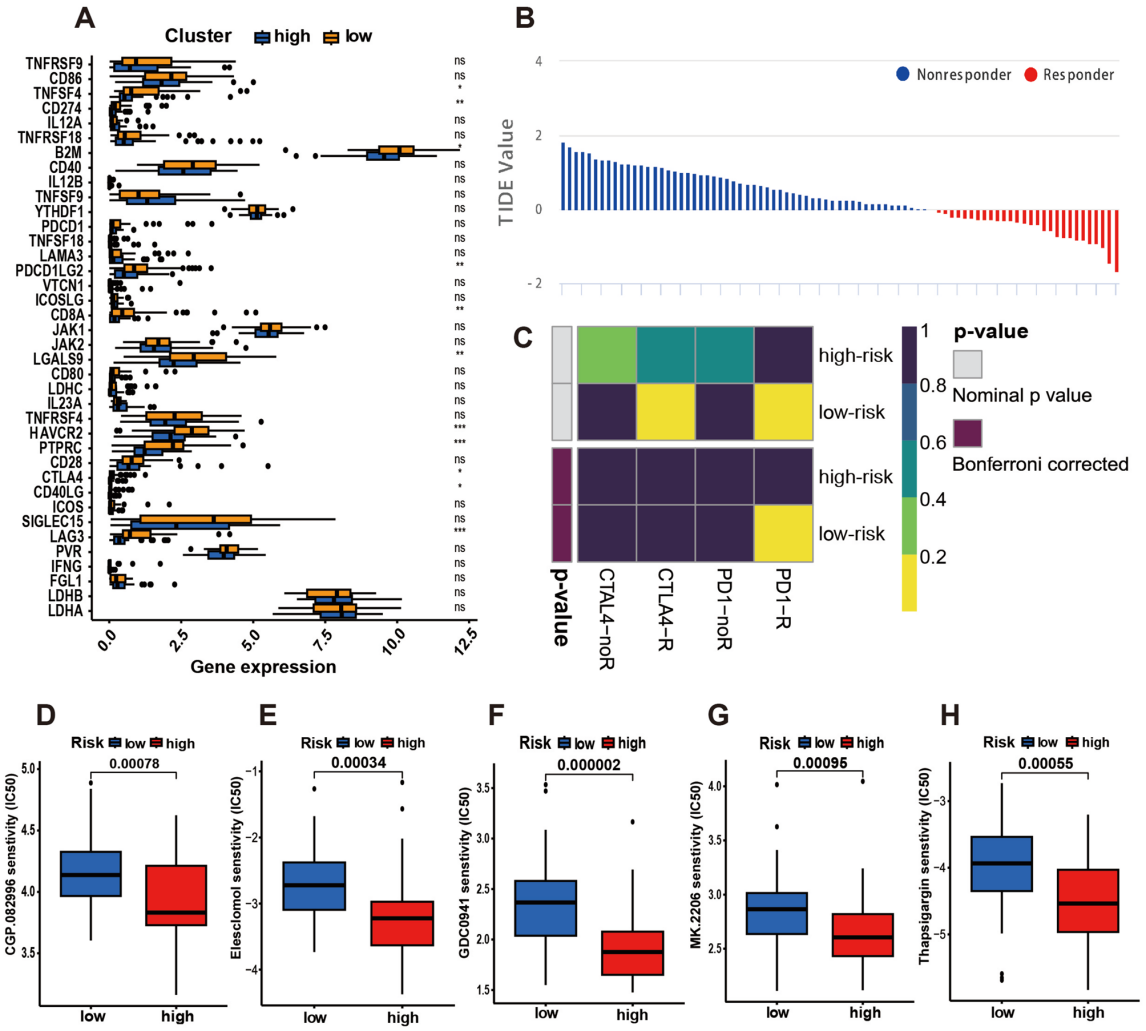
Prediction for immunotherapy and drug sensitivity analysis. (**A**) Boxplots depict the degree of expression of the 38 immune checkpoint molecules in the two risk subgroups. (**B**) TIDE value and the effect of immunotherapy of patients with OS. (**C**) According to submap analysis, the high-risk subgroup may benefit more from anti-PD-1 therapies. (**D–H**) Estimated IC50 indicated that 5 drugs were sensitive to patients in the high-risk group.

We found several molecules that are significant in tumor initiation and treatment by immune checkpoint analysis, such as lymphocyte-activation gene 3 (LAG3), PTPRC, HAVCR2, B2M, LDHA, and LDHB, which may be used as a reference for tumor immunotherapy (Fig. 6A). According to the analysis, the predicted treatment effects of anti-CTLA4 and anti-PD-1 therapy for the high- and low-risk groups are presented in Fig. 6B,C. Five drugs were demonstrated to be effective in the high-risk group in the drug sensitivity analysis: CGP.082996, elesclomol, pictilisib, MK.2206, and thapsigargin (Fig. 6D–H).

### In vitro JTB knockdown experiments

Figure 3A shows that OS tissue had a much higher level of JTB expression than normal tissue. Between the low and high JTB expression groups, there was a notable difference in the overall survival rate. Additionally, no research or reports on OS and JTB were discovered in a search of published literature. Thus, we decided to focus our experiments on PPS-OS-related genes specifically on JTB.

**Figure 7:**
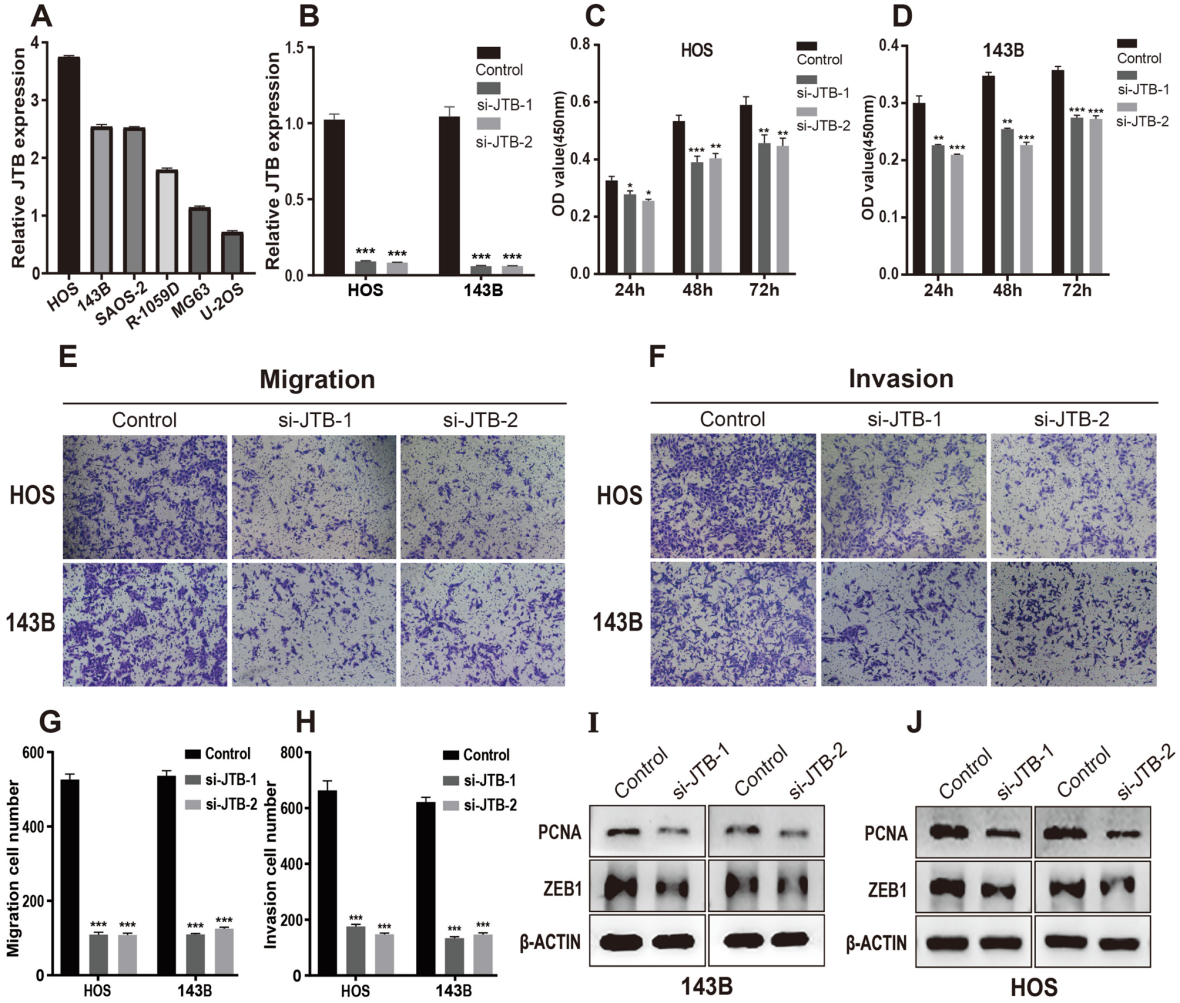
Verification of the silencing effect. (**A**) Relative expression of JTB in six OS cell lines. (**B**) The expression levels of JTB in HOS and 143B were detected by RT-qPCR after knocking down with siRNA. (**C,D**) Cellular viability (%) of CCK-8 experiment in HOS (**C**) cells and 143B (**D**) cells. (**E,F**) Representative imaging of migration assays (**E**) and invasion assays (**F**) after silencing with JTB-1 and JTB-2 in HOS and 143B cells. (**G,H**) Cell counts for migration assays (**G**) and invasion assays (**H**) after silencing with JTB-1 and JTB-2 in HOS and 143B cells. (**I,J**) 143B (**I**) and HOS (**J**) cells were transfected with si-NC/si-JTB followed by western blot analysis.

RT-qPCR revealed that HOS and 143B cells had higher JTB expression levels than the other cell lines (Fig. 7A). JTB was then knocked down in 143B and HOS cells using si-JTB-1 and -2, and the results were noticeable (Fig. 7B). The CCK-8 assay showed that the survivability of HOS and 143B cells after JTB knockdown was decreased compared with that of negative control cells (Fig. 7C,D). Then, the cell transwell experiment demonstrated that the number of 143B and HOS cells penetrating the micropore membrane was significantly decreased after silencing JTB, indicating that OS cell capacity for migration and invasion was impaired when JTB was silenced (Fig. 7E,F). Finally, the results of Western blotting demonstrated that si-JTB-1 and si-JTB-2 can effectively decrease JTB protein expression in 143B and HOS cells (Fig. 7I,J).

## Discussion

Over the past 30 years, the 5-year survival rate of patients with OS has improved with a combination of radiotherapy, chemotherapy and surgery^26^. However, metastatic or drugresistant OS continues to pose a challenge^27^. For refractory OS, new therapeutic targets are urgently needed for clinical application.

Previous evidence has shown that the heterogeneity of tumor cells reflects differences in the biological behavior of tumor cells. To date, studies on cancer, especially studies focused on identifying the interplay between ITH and TME in different subclones, have benefited greatly from scRNA-seq technology^28^. Therefore, further understanding of the heterogeneity between OS cells and its mechanism of action based on scRNA-seq data may provide clues for developing novel therapies. We conducted a functional enrichment analysis of genes related to PPS-OS in this study, and the results were mainly related to the critical step and molecular machine of protein synthesis. A transgenic mouse model of B-cell Burkitt lymphoma driven by Eµ-Myc revealed a possible role for hyperactive ribosomal biogenesis in the progression of cancer^29^. Both the increased efficiency of protein synthesis and the decreased fidelity of translation are associated with aberrant ribosome biogenesis, which may lead to tumorigenesis^30–32^. Therefore, the identification of genes associated with PPS-OS may aid in the study of the heterogeneity and underlying mechanisms of OS cells, which may provide a foundation for improving OS diagnosis and treatment^33^.

First, we assessed scRNA-seq data from public databases and annotated seven cell subtypes. Among them, PPS-OS indicated the worst prognosis of patients. Next, by LASSO regression analysis, we constructed a model based on 6 genes. The ROC analysis displayed the accuracy with which the model predicts prognosis. In the nomogram we constructed, metastasis status had the highest weighted score, followed by the risk score and tumor primary site. Nomograms, which are multivariable regression models that generate individual numerical probabilities of clinical events by integrating several prognostic and determinant variables^34^, have been widely used in various studies^35^. The calibration curves demonstrated the nomogram’s prognosis prediction efficiency in a straightforward manner, confirming the accuracy of our model.

The ratio of malignant cells among all cells in a tumor tissue specimen was recognized as the tumor purity, and it was directly correlated with a poor prognosis^36^. The ESTIMATE algorithm is a novel algorithm relying on gene expression data, and the infiltration of nontumor cells from cancer samples determines the stromal score and immune score. The transcriptional data were then used to determine tumor purity based on immune score and stromal score^37^. In accordance with our research, lower overall survival, stromal score, immune score, and ESTIMATE score, as well as higher tumor purity, were observed in the high-risk group. This partially explains why patients who were classified into the high-risk group had worse survival rates.

Tumor-infiltrating immune cells (TIICs) make up the majority of the complex mixture of cells in the tumor immune microenvironment (TIME)^38^. Numerous studies have demonstrated that TIICs play a crucial role in the occurrence, recurrence and metastasis of OS^39,40^. Understanding TIICs is crucial in treatment optimization and prognosis improvement in patients^41^. Our research indicates that B cells and gamma delta T cells (Tgd, γδ T cells) were higher in the high-risk group. B cells and γδ T cells are important tumor immune cells in vivo, especially in tumor immunity. They would, however, evolve into immunosuppressive cells to promote carcinogenesis^42–44^. After mitogen activation, γδ T cells can be stimulated by autologous B cells^45^. Subsequently, γδ T cells are able to influence B-cell function by suppressing the secretion of IgG^46^. Disruption of the immune balance maintained by these cells leads to inflammation and promotes tumor immune escape^47^. Studies have also shown that the exosomes released by OS have been found to contain an immunomodulatory substance that targets T cells, which reduces T cell activity and promote the regulatory phenotype T^48^. Experiments in mouse models have shown that B cells can inhibit the antitumor T cell response to promote tumorigenesis^49,50^. Therapies that boost the antitumor response mediated by innate immune cells, including T cells, are beneficial for OS patients^51^. Thus, this immunological profile may contribute to the malignant features of OS.

TIDE is an algorithm for predicting the immune checkpoint blockade (ICB) response in cancer by predicting tumor immune evasion activity based on specific expression signatures (T-cell dysfunction and T-cell exclusion)^52^. As such, its predictive value for the response to immune checkpoint inhibitors is evident^53^. Based on the two reliable targets CTLA-4 and PD-1, a variety of targeted agents have been approved for the treatment of different cancers^54^. The US Food and Drug Administration has authorized pembrolizumab (PD-1) and ipilimumab (CTLA-4) for the treatment of metastatic melanoma^55^. In recent years, CTLA-4 and PD-1/PD-L1 blockade have exhibited great application potential for OS immunotherapy^56,57^. The results we obtained imply that low-risk patients identified by our model may respond well to PD-1 and CTLA4 blockade.

For malignant tumors, chemotherapy is a common treatment, but because of its poor bioavailability and lack of targets, its efficacy still confronts severe clinical challenges. Previous studies have suggested that tumor-derived or tumor-associated exosomes are critical in modulating tumor drug resistance^58^. According to our analysis and prediction, five drugs were obtained: elesclomol, pictilisib, MK.2206, and thapsigargin. Among them, MK-2206, an AKT inhibitor, could suppress the observed decrease in sensitivity to chemotherapeutic agents induced by exosomes^59^. Based on the results of our study, a combined strategy utilizing immunotherapy and targeted drugs may serve as a new approach for the treatment of OS^51^.

JTB is a gene located on human chromosome 1q21^60^. While JTB is widely expressed in normal cells, it has been discovered that cancer cells overexpress it^61^. Numerous investigations have demonstrated that JTB can promote the proliferation, invasion and metastasis of a variety of cancer cells^62^. Jayathirtha et al. explored the impact of downregulation of JTB expression in MCF-7 breast cancer cells, laying the foundation for its potential application as a biomarker in breast cancer^63^. Sanford et al. concluded that JTB in myeloid malignancies was associated with treatment resistance and poor survival^64^. However, its role in OS is unknown. In our study, the analysis of OS data and in vitro experimental results suggested that JTB knockdown may prevent OS cells from proliferating, migrating, and invading and may affect protein expression. These findings deepen our understanding of the biological basis of OS and indicate that JTB may become a novel therapeutic target for OS in humans.

## Conclusion

In this study, the collected single-cell OS data were divided into seven subtypes, and the PPS-OS subtype was further investigated. Based on the six PPS-OS correlated genes, we constructed a model with the ability to predict the prognosis of OS patients. Moreover, the difference in the TIME features of OS patients in different risk groups OS were presented. Corresponding functional experiments demonstrated that the capacity of OS cells to migrate and invade was impaired when JTB was silenced. Although this study achieved ideal positive results, further studies are also warranted. To conclude, our findings may contribute to a strategy for predicting prognosis and facilitate the identification of novel therapeutic targets for OS patients.

### Supplementary Information

Supplementary Legends.

Supplementary Figure 1.

Supplementary Figure 2.

Supplementary Figure 3.

Supplementary Table 1.

Supplementary Table 2.

## Data Availability

All data included in this study are available upon request by contact with the corresponding author.
