# Supplementary material for: Identification of a pro-protein synthesis osteosarcoma subtype for predicting prognosis and treatment
Source: Sci Rep. 2024 Jul 16;14:16475. doi: 10.1038/s41598-024-67547-z (PMC11252356; doi:10.1038/s41598-024-67547-z)
Supplement: Supplementary file 1 — Supplementary Legends. [file 41598_2024_67547_MOESM1_ESM.doc]

**Supplementary Table1** 16 Cell subtypes and their genes, it also includes P values of genes, average log2FC, adjusted P value, etc.

**Supplementary Table2** 7 OS Cell subtypes and their genes, it also includes P values of genes, average log2FC, adjusted P value, etc.

**Supplementary Figure1** The GO functional analysis in distinct cell subtypes of OS.

**Supplementary Figure2** Kaplan–Meier survival curves and ROC curves of the six cell subtypes except PPS-OS subtype. Red represents the high risk group, whereas blue represents the low risk group.

**Supplementary Figure3** Full-length gels and blots in western blot. 143B (A) and HOS (B) cells were transfected with si-NC/si-JTB followed by western blot analysis. The antibodies used in this experiment included anti-PCNA (Proliferating Cell Nuclear Antigen), anti-ZEB1 (Zinc finger E-box-binding homeobox 1) and anti-β-actin.
