# Supplementary figures and images for: Identification of a pro-protein synthesis osteosarcoma subtype for predicting prognosis and treatment
Source: Sci Rep. 2024 Jul 16;14:16475. doi: 10.1038/s41598-024-67547-z (PMC11252356; doi:10.1038/s41598-024-67547-z)

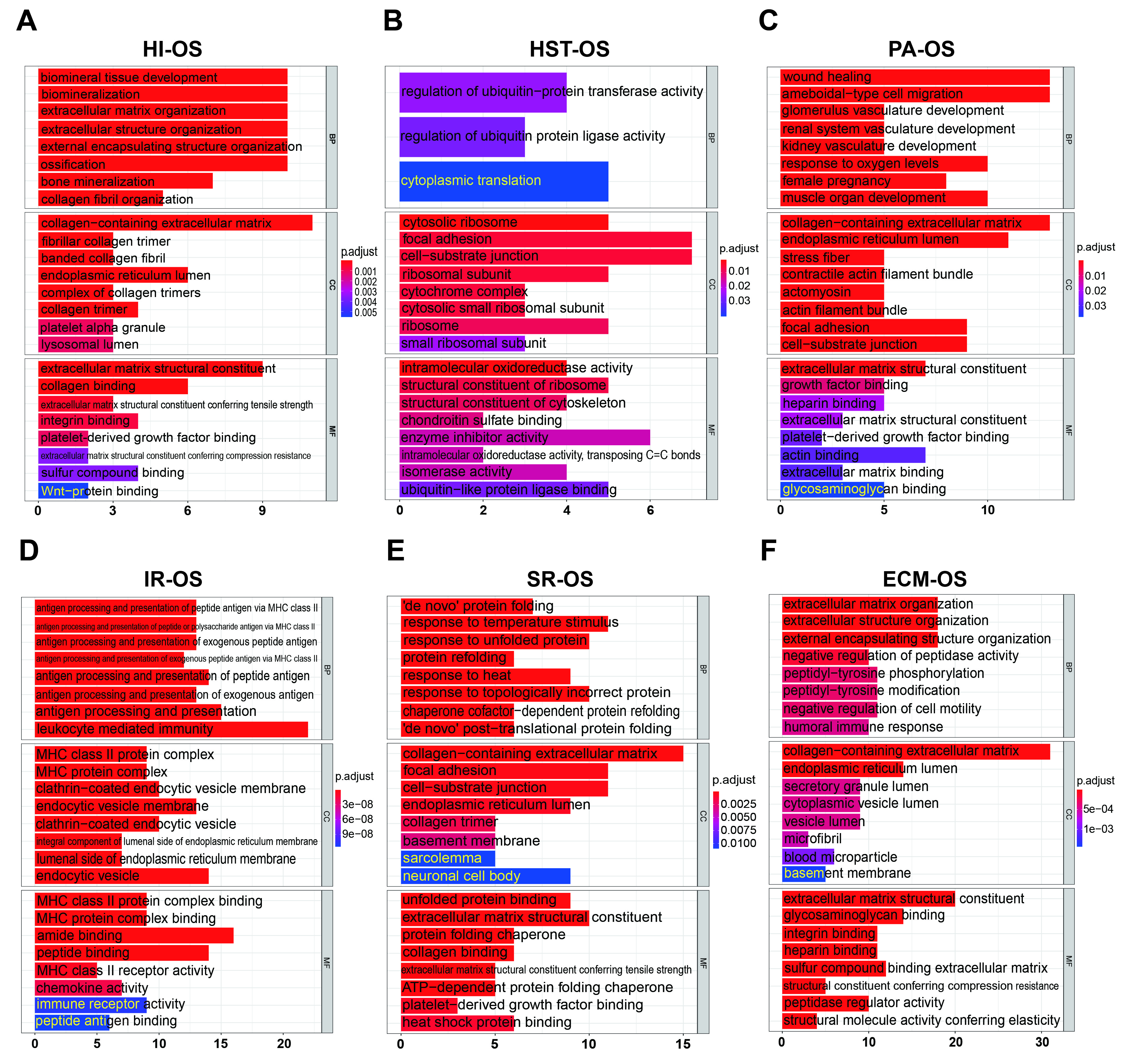

Supplement: Supplementary file 2 — Supplementary Figure 1. [file 41598_2024_67547_MOESM2_ESM.jpg]

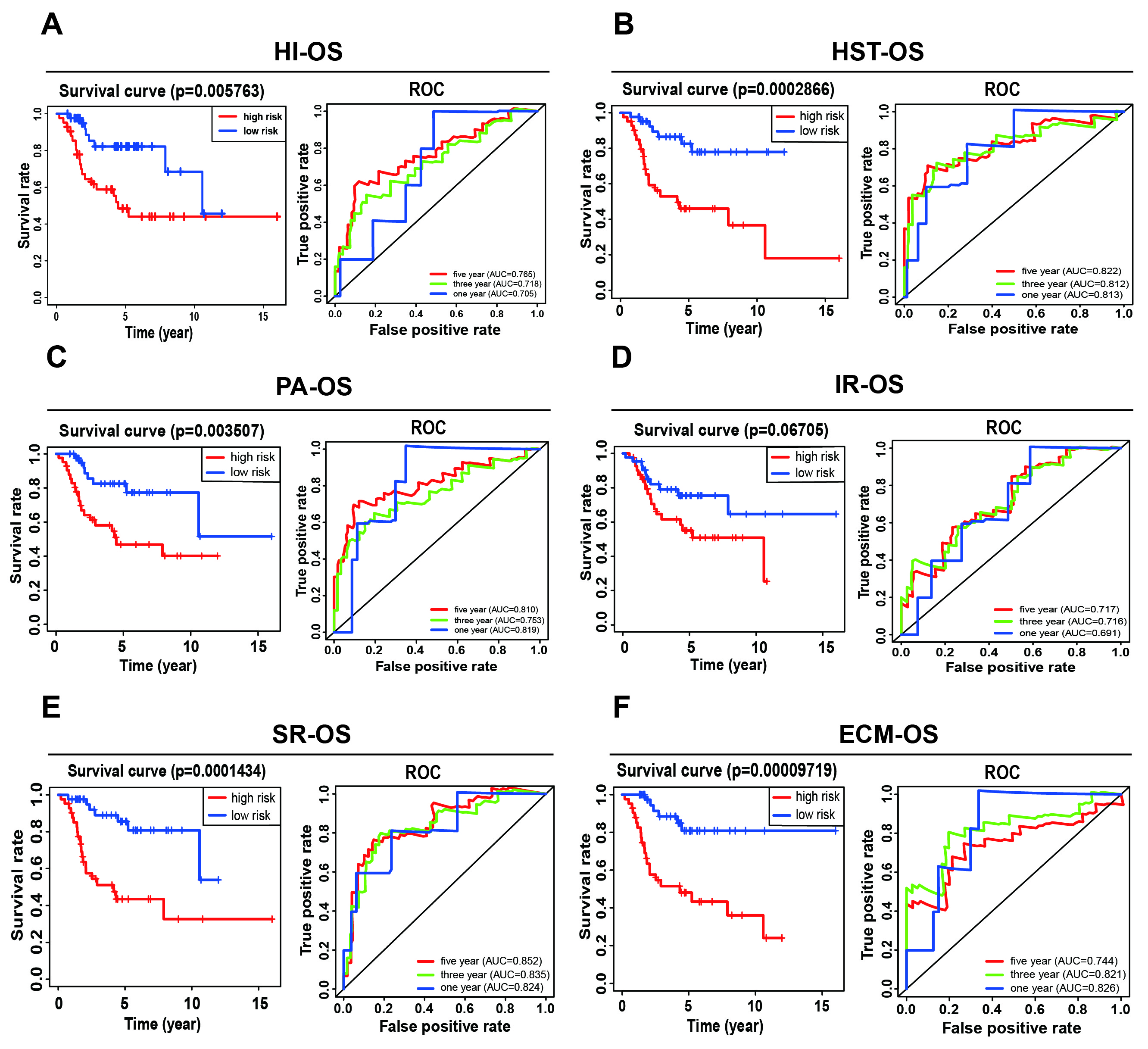

Supplement: Supplementary file 3 — Supplementary Figure 2. [file 41598_2024_67547_MOESM3_ESM.jpg]

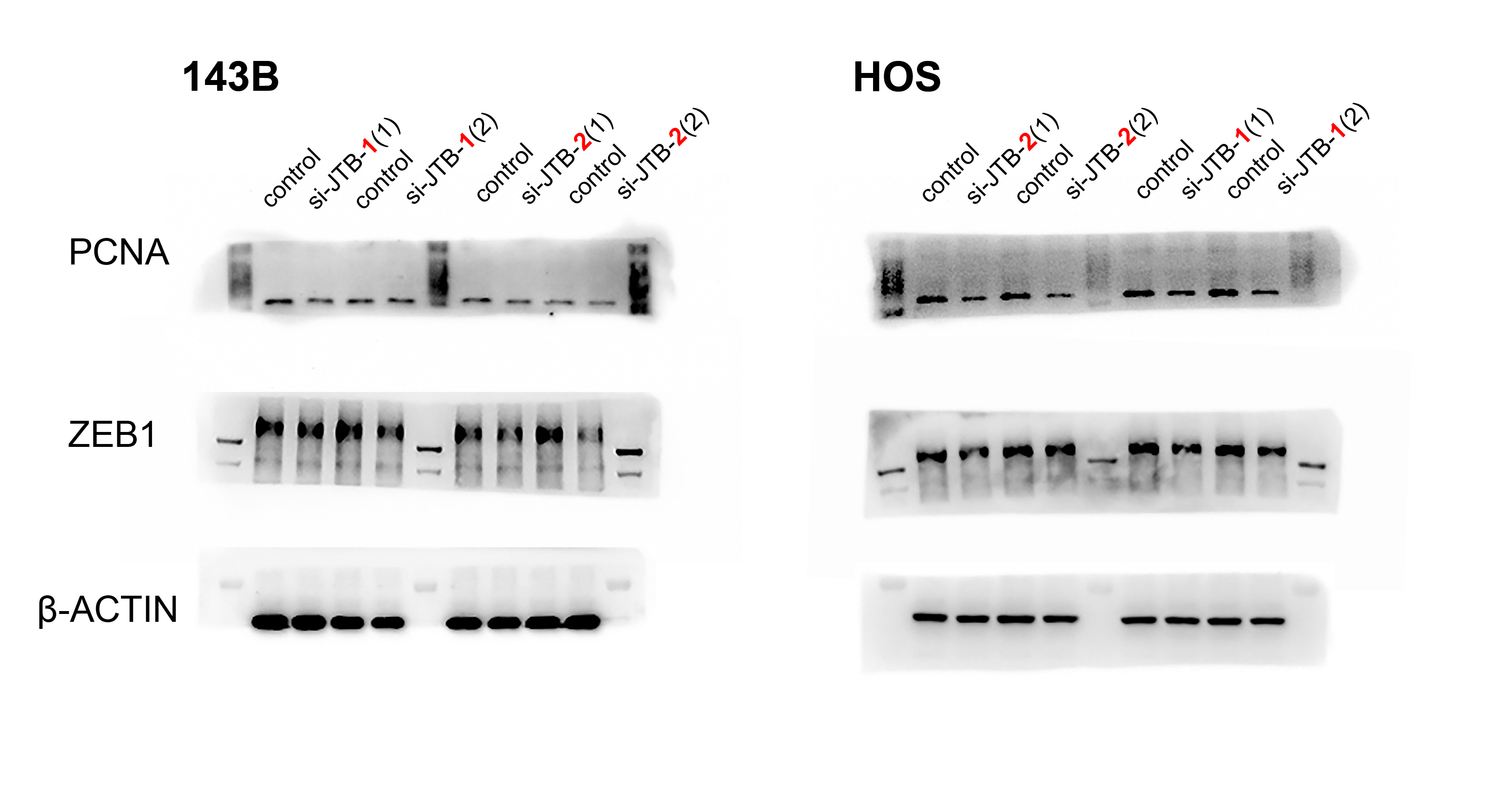

Supplement: Supplementary file 4 — Supplementary Figure 3. [file 41598_2024_67547_MOESM4_ESM.jpg]
